# Sichere R0-Resektion zur Vermeidung der adjuvanten Therapieeskalation beim Oropharynxkarzinom

**DOI:** 10.1007/s00106-020-00932-y

**Published:** 2020-09-25

**Authors:** N. Mansour, C. Backes, C. Becker, B. Hofauer, A. Knopf

**Affiliations:** 1 Klinik für Hals, Nasen- und Ohrenheilkunde, Universitätsklinikum Freiburg, Killianstr. 5, 79106 Freiburg, Deutschland; 2 Klinik und Poliklinik für Hals‑, Nasen- und Ohrenheilkunde, Klinikum rechts der Isar, Technische Universität München, München, Deutschland

**Keywords:** Überlebensanalyse, Therapieergebnis, Kopf-Hals-Neoplasien, Exzisionsränder, Adjuvante Radiochemotherapie, Survival analysis, Treatment outcome, Head and neck neoplasms, Margins of excision, Adjuvant chemoradiotherapy

## Abstract

**Hintergrund:**

Der Resektionsstatus ist ein Prädiktor bei Patienten mit Oropharynxkarzinomen (OPSCC) für die Überlebensrate (ÜR) und das rezidivfreie Überleben (RFÜ). TNM-Status, extrakapsuläres Wachstum im Lymphknoten (ECE) und der Resektions(R)-Status des Primärtumors entscheiden über die adjuvante Therapie, wobei der R‑Status unmittelbar durch den Kopf-Hals-Chirurgen beeinflussbar ist. Ziel dieser Arbeit ist die Evaluation des Einflusses des R‑Status auf Therapieentscheidungen, RFÜ und ÜR.

**Material und Methoden:**

Es wurden alle Patienten mit Plattenepithelkarzinom des Oropharynx, die im Zeitraum von 2001–2011 operiert wurden, in die Auswertung eingeschlossen. Retrospektiv wurden klinische Parameter, Überlebensdaten, histologische Daten wie ECE, R‑Status und Tumorausdehnung erhoben sowie analysiert.

**Ergebnisse:**

Es wurden 208 Patienten in die Studie eingeschlossen. Patienten mit R0-Resektion zeigten ein mittleres RFÜ/ÜR von 89/87 Monaten. Dieses sank signifikant bei Patienten mit R1-Resektion (65/65 Monate), R2-Resektion (38/33 Monate) sowie Rx-Resektion (59/45 Monate; *p* = 0,036/*p* = 0,001). Bei Patienten mit ECE und R1-Resektion, aber auch mit R0-Resektion durch Nachresektion und Rx-Status erfolgte eine adjuvante Therapieeskalation.

**Schlussfolgerung:**

Unsichere Resektionsränder reduzieren das RFÜ und die ÜR. Daher sollte bei einer chirurgischen Therapie immer eine R0-Resektion möglichst am Hauptpräparat angestrebt werden, um eine adjuvante Therapieeskalation wegen eines unklarer R‑Status zu vermeiden.

## Abstract (en)

**Background:**

Resection margins in patients with oropharyngeal squamous cell carcinoma (OPSCC) are a predictive marker for overall survival (OS) and recurrence-free interval (RFI). Adjuvant therapy is influenced by TNM status, extracapsular extension (ECE), and resection margin status (R) of the primary tumor. The R status can be directly influenced by the head and neck surgeon. The aim of the current study was to evaluate the impact of R status on treatment decisions, RFI, and OS.

**Materials and methods:**

All patients with OPSCC who underwent surgery (with/without adjuvant therapy) between 2001 and 2011 were enrolled. Clinical data, survival parameters, histologic data such as ECE, resection margin status, and tumor size were retrospectively collected and analyzed.

**Results:**

A total of 208 patients were enrolled. Survival parameters showed that patients with microscopically clear (R0) resection margins had an RFI/OS of 89/87 months. These values decreased in patients with R1 (65/65 months), R2 (38/33 months), and Rx (unclear) resections (59/45 months; *p* =  0.036/*p* = 0.001). In patients with ECE and R1 resection, but also in those with R0 resections achieved by follow-up resection and those with Rx resections, adjuvant therapy was escalated.

**Conclusion:**

Unclear resection status reduces OS and RFI in patients with OPSCC. Therefore, in surgical therapy, clear resection status in the first pass should be strived for to avoid escalation of adjuvant therapy due to an unclear R status.

Kopf-Hals-Tumoren sind weltweit die siebthäufigsten krebsassoziierten Todesursachen [1]. Bei den Kopf-Hals-Tumoren handelt es sich in 90 % der Fälle um Plattenepithelkarzinome („squamous cell carcinoma“, SCC) [2, 3]. Obwohl Larynxkarzinome an Inzidenz abnehmen, steigt die Rate an Neuerkrankungen mit Oropharynxkarzinomen (OPSCC) an. Dies ist der Tatsache geschuldet, dass die Aufklärung über die Schädlichkeit der üblichen Risikofaktoren wie Nikotin- und Alkoholabusus Früchte trägt, aber insbesondere die mit humanen Papillomaviren (HPV-)assoziierten OPSCC zunehmen [4, 5]. OPSCC werden oft in fortgeschrittenem Tumorstadium diagnostiziert. Bei chirurgisch resezierbaren Tumoren bedarf es dennoch in vielen Fällen einer adjuvanten Therapie. Indikationen hierfür sind nicht nur fortgeschrittener Tumor- (T-) und Nodal- (N-)Status, sondern auch extrakapsuläres Wachstum am Lymphknoten („extracapsular extension“, ECE) sowie der Resektions- (R-)Status. Bei Nachweis von ECE und R1-Status nach chirurgischer Therapie haben Bernier et al. gezeigt, dass eine adjuvante Therapieeskalation mittels Radiochemotherapie im Vergleich zur alleinigen Strahlentherapie einen Überlebensvorteil bietet [6]. Dem stehen Spättoxizitäten der Therapieverfahren gegenüber, die insbesondere bei der Radio(chemo)therapie noch nach Jahren auftreten können und nicht absehbar sind.

Während insbesondere ein mikroskopisches ECE oft trotz hochmoderner bildgebender Diagnostik präoperativ nicht antizipiert werden kann, ist insbesondere der R‑Status durch den Chirurgen beeinflussbar, sodass eine möglicherweise bereits durch TN-Status indizierte adjuvante Therapie im Sinne einer Radiotherapie nicht unnötig eskaliert werden muss. In dieser Studie evaluierten die Autoren Patienten mit OPSCC im Hinblick darauf, ob und in welcher Weise der R‑Status im Rahmen der chirurgischen Therapie einen Einfluss auf Therapieentscheidungen, rezidivfreies Überleben (RFÜ) und Überlebensrate (ÜR) nimmt.

## Material und Methoden

Es wurden alle Patienten in die Studie eingeschlossen, die ein histologisch gesichertes Plattenepithelkarzinom des Oropharynx aufwiesen und einer chirurgischen Therapie mit oder ohne adjuvante Behandlung im Zeitraum von 2001 bis 2011 zugeführt wurden. Alter, Geschlecht, TNM-Status entsprechend der 7. Auflage der UICC (Union Internationale Contre le Cancer), die Differenzierung (Grading), die Therapiemodalitäten und Überlebensdaten (rezidivfreies Überleben, Tod/verloren in der Nachbeobachtung) wurden retrospektiv erhoben.

In der Histologie wurden der maximale Tumordurchmesser und die maximalen tumorfreien Absetzungsränder zirkumferent (an den Schleimhautabsetzungsrändern) und zur Tiefe (Wundgrund bzw. Absetzungsrand gegenüber der Schleimhautoberfläche) evaluiert. Zudem wurde untersucht, ob ein R0-Status bereits am Hauptpräparat oder erst durch Nachresektionen erreicht wurde. Eine Untersuchung auf extrakapsuläres Wachstum wurde bei allen Lymphknotenmetastasen durchgeführt.

Statistische Analysen wurden unter den Gruppen mittels χ^2^- und exaktem Test nach Fisher sowie t‑Test für unverbundene Stichproben durchgeführt. ANOVA („analysis of variance“) und Tukey-Tests wurden zur Analyse mehrerer Gruppen verwendet. Es wurden Korrelation zwischen T‑, N‑ und R‑Status berechnet und mittels Korrelationskoeffizient nach Pearson ausgedrückt. Überlebensraten und -kurven wurden berechnet, mittels Kaplan-Meier-Kurven dargestellt und Log-Rank-Tests durchgeführt. *p*-Werte <0,05 wurden als signifikant angesehen. Die statistischen Berechnungen wurden mittels SPSS (Fa. SPSS Inc., Chicago, IL, USA) durchgeführt.

## Ergebnisse

In die Auswertung wurden 208 Patienten eingeschlossen. Davon wurde bei 129 Patienten eine R0-Resektion am Hauptpräparat erreicht, bei 17 ein R0-Status durch Nachresektionen, bei 33 wurde ein R1-Status erreicht, bei 2 Patienten ein R2-Status und bei 17 Patienten ein Rx-Status (Tab. 1). Da die Gruppe der Patienten mit R2-Status so klein war, wurde für diese Gruppe nur die Kaplan-Meier-Kurve für die ÜR und das RFÜ berechnet und der Log-Rank-Test zur Berechnung von Signifikanzen zu den anderen Gruppen angewandt. Aus weiteren Analysen wurden diese beiden Patienten ausgeschlossen. Das mittlere Alter der gesamten Patientengruppe reichte von 56 bis 59 Jahren ohne signifikanten Unterschied in den Gruppen (*p* = 0,39). Auch die Verteilung in Bezug auf das Geschlecht zeigte keine signifikanten Unterschiede in den Gruppen (*p* = 0,10). Die Verteilung des T‑ und M‑Status sowie des Gradings wies zwischen den Gruppen keine signifikanten Unterschiede auf (*p* = 0,26; *p* = 0,75; *p* = 0,27). Lediglich für den N‑Status zeigte sich zwischen den Gruppen ein signifikanter Unterschied (*p* = 0,049). Patienten mit R1- oder Rx-Status zeigten tendenziell eher eine fortgeschrittene zervikale Lymphknotenmetastasierung (N2a‑, N2b- oder N2c-Status), während Patienten mit R0-Status am Hauptpräparat oder durch Nachresektion zu einem N0- und N1-Status tendierten (Tab. 1).

**Table 1:** 

	R0 HP	R0 NR	R1	Rx	*p*-Wert
*n *(Anzahl)	129	17	33	27	–
Alter (Jahre)					0,39
MW ± SD	59 ± 9	56 ± 11	59 ± 10	56 ± 10	–
Geschlecht, *n* (%)					0,10
Männlich	116 (90)	14 (82)	25 (76)	20 (74)	–
Weiblich	13 (10)	93(18)	8 (24)	7 (26)	–
T‑Stadium, *n* (%)					0,26
T1	51 (40)	7 (41)	11 (33)	10 (37)	–
T2	57 (44)	6 (35)	14 (42)	11 (41)	–
T3	14 (11)	4 (24)	3 (9)	4 (15)	–
T4	7 (5)	0	5 (15)	2 (7)	–
N‑Stadium, *n* (%)					0,049*
N0	45 (35)	7 (41)	9 (27)	5 (19)	–
N1	32 (25)	3 (18)	2 (6)	6 (22)	–
N2a, b	51 (40)	7 (41)	19 (58)	12 (44)	–
N2c	9 (7)	0	2 (6)	4 (15)	–
N3	1 (1)	0	1 (3)	0	–
M‑Stadium, *n* (%)					0,75
M0	128 (99)	17 (100)	32 (97)	27 (100)	–
M1	1 (1)	0	1 (3)	0	–
Grading, *n* (%)					0,27
G1	2 (2)	1 (6)	0	0	–
G2	61 (47)	9 (53)	7 (21)	10 (37)	–
G3	65 (50)	6 (35)	26 (79)	17 (63)	–
G4	0	1 (6)	0	0	–
Gx	1 (1)	0	0	0	–

*MW* Mittelwert, *SD* Standardabweichung

In der Korrelation zwischen T‑ und N‑Status zum R‑Status zeigte sich keine Korrelation zwischen T‑ und R‑Status (r = 0,134) und eine sehr schwache Korrelation zwischen N‑ und R‑Status (r = 0,190).

Da bei OPSCC ein höherer T‑Status auch durch Infiltrationen von benachbarten Kompartimenten entsteht, wurde anhand der histologischen Präparate die maximale Tumorausdehnung (in Millimetern, mm) analysiert sowie die tumorfreien Resektionsränder (in mm). Die maximale Tumorgröße war im Mittel zwischen 24 und 26 mm ohne signifikanten Unterschied zwischen den Gruppen (*p* = 0,82). Der  kleinste tumorfreie Absetzungsrand lag zwischen 3 und 4 mm und war ebenfalls nicht signifikant (*p* = 0,53). Auch die Lokalisation des kleinsten tumorfreien Absetzungsrandes, zirkumferent, zur Tiefe oder beides, war nicht signifikant (*p* = 0,12). Gerade in der Gruppe der R1-Resektionen zeigte sich jedoch der kleinste tumorfreie Absetzungsrand v. a. zur Tiefe (Tab. 2).

**Table 2:** 

	R0 HP	R0 NR	R1	*p*-Wert
*n* (Anzahl)	129	17	33	–
Größter Durchmesser am Primärtumor (mm)	24 ± 14	26 ± 14	26 ± 13	0,82
Kleinster tumorfreier Rand (mm)	3 ± 2	4 ± 3	–	0,53
Kleinster tumorfreier Rand, Lokalisation	–	–	–	0,12
Zirkumferenz	54 (42)	7 (41)	6 (18)	–
Tiefe Absetzung	57 (44)	5 (29)	19 (58)	–
Beides	18 (14)	5 (29)	8 (24)	–

**Figure 1:**
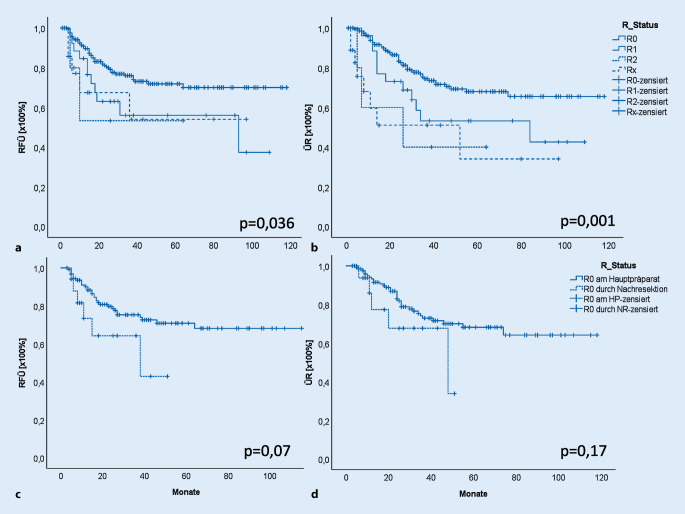

Die Analysen von ÜR und RFÜ zeigten signifikante Unterschiede zwischen den Gruppen. Patienten mit R0-Status zeigten ein mittleres Überleben von 87 Monaten. Bei Patienten mit R1-Resektion war dieses nur noch 65 Monate, bei Patienten mit R2-Resektion sogar nur 33 Monate und mit Rx-Resektion 45 Monate (*p* = 0,001, Abb. 1b). Entsprechend der ÜR zeigte sich bei den Patienten mit R0-Resektion das beste RFÜ von 89 Monaten, bei Patienten mit R1-Resektion ein RFÜ von 65 Monaten, mit R2-Resektionen von 38 Monaten und Rx-Resektion von 59 Monaten (*p* = 0,036; Abb. 1a). Die mittlere Nachbeobachtungszeit betrug 61 Monate.

Es wurden Subgruppenanalysen bei den Patienten mit R0-Resektion durchgeführt. Hier wurde zwischen einer R0-Resektion am Hauptpräparat und einer R0-Resektion durch Nachresektion unterschieden. Es zeigte sich, dass für die ÜR kein signifikanter Unterschied zwischen den Subgruppen vorlag (*p* = 0,17; Abb. 1d), aber eine Tendenz beim RFÜ. Patienten mit R0-Resektion am Hauptpräparat haben mit 87 Monaten ein längeres RFÜ als Patienten mit R0-Resektion durch Nachresektion mit 33 Monaten (*p* = 0,07; Abb. 1c).

Bezüglich der therapeutischen Strategien wurden Analysen durchgeführt, um zu evaluieren, ob das therapeutische Management Einfluss auf den R‑Status nahm. Die Art der chirurgischen Resektion und das Ausmaß der Neck-Dissection zeigten keinen signifikanten Unterschied bei den Patienten mit verschiedenem R‑Status (*p* = 0,11; *p* = 0,14; Tab. 3). Das extrakapsuläre Wachstum ist wesentliches Kriterium für eine adjuvante Radiochemotherapie. Ein extrakapsuläres Wachstum zeigte sich bei 10 % der Patienten mit R0-Resektion am Hauptpräparat, bei 12 % mit R0-Status durch Nachresektion, bei 18 % mit R1-Resektion und 26 % bei Rx-Resektion (*p* = 0,11; Tab. 3). Im Gegenzug dazu wurde eine adjuvante Radiochemotherapie bei 31 % bei Patienten mit R0-Resektionen am Hauptpräparat und 47 % mit R0-Resektion durch Nachresektion durchgeführt. Bei Patienten mit R1-Resektion wurde in 64 % und bei Patienten mit Rx-Resektion in 67 % der Fälle eine adjuvante Radiochemotherapie durchgeführt (*p* < 0,0001; Tab. 3). Die Unterschiede in der adjuvanten Therapie wurden weiter evaluiert, um die Therapieeskalation aufgrund des R‑Status beurteilen zu können. Eine Therapieeskalation in Sinne einer Radio- bzw. Radiochemotherapie erfolgte bei 17 % bei Patienten mit R0-Status am Hauptpräparat, bei 35 % bei Patienten mit R0-Resektion durch Nachresektion, bei 45 % bei Patienten mit R1-Resektion und bei 33 % bei Patienten mit Rx-Resektion. Dieser Unterschied war statistisch signifikant (*p* = 0,009; Tab. 3).

**Table 3:** 

	R0 HP	R0 NR	R1	Rx	*p*-Wert
*n* (Anzahl)	129	17	33	27	–
Chirurgie des Primärtumors, *n* (%)					0,11
Transorale Resektion	81 (63)	12 (71)	23 (70)	19 (70)	–
Transmandibuläre Resektion	27 (21)	2 (12)	8 (24)	4 (15)	–
Pharyngotomie	16 (12)	1 (6)	1 (3)	3 (11)	–
Pharyngo‑/Laryngektomie	2 (2)	–	1 (3)	–	–
Andere Techniken	1	1 (6)	–	1 (4)	–
Partielle Mandibulektomie	2 (2)	1 (6)	–	–	–
Neck-Dissection, *n* (%)					0,14
Keine	16 (12)	1 (6)	5 (15)	3 (11)	–
Ipsilateral	52 (40)	9 (53)	16 (49)	9 (33)	–
Beidseits	61 (47)	7 (41)	12 (36)	15 (56)	–
ECE-Status, *n* (%)					0,11
Negativ	116 (90)	15 (88)	27 (82)	20 (74)	–
Positiv	13 (10)	2 (12)	6 (18)	7 (26)	–
Adjuvante Therapie, *n* (%)					<0,0001*
Op. allein	30 (23)	6 (35)	1 (3)	2 (7)	–
Op. + RT	59 (46)	3 (18)	11 (33)	7 (26)	–
Op. + RCT	40 (31)	8 (47)	21 (64)	18 (67)	–
Adjuvante Therapieeskalation bei unzureichendem R‑Status, *n* (%)					0,009*
Keine	107 (83)	11 (65)	18 (55)	18 (67)	–
RT	5 (4)	1 (6)	32 (6)	2 (7)	–
RCT	17 (13)	5 (29)	13(39)	7 (26)	–

*ECE* extrakapsuläres Wachstum am Lymphknoten („extracapsular extension“),* RCT* Radiochemotherapie, *RT* Radiotherapie

## Diskussion

Im Management des OPSCC muss eine Balance gewahrt werden zwischen ausreichender Radikalität bzw. Intensität der Therapie zugunsten eines langen RFÜ sowie einer langen ÜR und einer bestmöglichen posttherapeutischen Funktionalität mit guter Lebensqualität für die Patienten. In unterschiedlichen Studien wurde gezeigt, dass bei Kopf-Hals-Karzinomen nicht nur TNM-Status, sondern auch ECE in Lymphknoten und positive Absetzungsränder einen Einfluss auf ÜR und RFÜ haben [7–10]. In den späten 1990er-Jahren wurde in wegweisenden Studien nachgewiesen, das ECE und positive Absetzungsränder einer Therapieeskalation bedürfen. Durch eine adjuvante Radiochemotherapie im Vergleich zur alleinigen adjuvanten Strahlentherapie konnte das RFÜ und die ÜR verbessert werden [6, 11, 12]. Im Gegensatz dazu steht, dass es zu Langzeitspätschäden mit Funktionseinbußen nach kombinierter simultaner Radiochemotherapie kommt, die die Lebensqualität der Patienten maßgeblich beeinflussen [13–17]. Die Toxizität nach Radio(chemo)therapie wird in akut (bis 90 Tage nach Therapieende) und chronisch (ab dem 91. Tag) unterteilt. Allerdings gibt es kein klares Ende für die Spätschäden nach definitiver oder adjuvanter Radio(chemo)therapie.

Bisher ist insbesondere das mikroskopische ECE nicht ausreichend präoperativ, etwa bildmorphologisch, antizipierbar. Lediglich auf den R‑Status kann der Kopf-Hals-Chirurg bis zu einem gewissen Grad Einfluss nehmen. In dieser Studie konnten die Autoren bestätigen, dass positive Absetzungsränder bei OPSCC mit einem deutlichen Einbruch im RFÜ von 89 Monaten bei R0-Resektion auf 65 Monate bei R1-Resektion und 38 Monate bei R2-Resektion einhergehen. Ähnliches galt für die ÜR. Hier reduzierte sich die ÜR von 87 Monaten bei R0-Resektion auf 65 Monate bei R1-Resektion und 33 Monate bei R2-Resektion. In der Subgruppenanalyse zwischen R0-Resektion am Hauptpräparat und R0-Resektion durch Nachresektionen zeigte sich sogar eine Tendenz im RFÜ zugunsten der R0-Resektion am Hauptpräparat. Patienten, bei denen im Hauptpräparat bereits eine R0-Resektion erfolgen konnte, wiesen ein RFÜ von 88 Monaten auf, während Patienten, die eine R0-Resektion durch Nachresektion erlangten, lediglich ein RFÜ von 33 Monaten zeigten und somit ein ähnlich schlechtes RFÜ aufwiesen wie Patienten mit R2-Resektion. Um möglicherweise den R‑Status präoperativ zu antizipieren, führten die Autoren Korrelationsanalysen durch. Hier zeigte sich in dieser Kohorte keine Korrelation zwischen T‑ und R‑Status. Dies kann einem Bias durch die Wahl des Zugangs geschuldet sein. Lediglich der N‑Status zeigte eine sehr schwache Korrelation zum R‑Status. Bei hoher nodaler Last kann dies als zusätzliche Entscheidungshilfe in Zusammenschau aller Befunde und des Allgemeinzustands des Patienten bei der präoperativen Beratung für Chirurg und Patient fungieren.

Obwohl die tumorfreien Resektionsränder am Hauptpräparat durchaus knapp waren, war die Faktenlage zur Entscheidung über eine adjuvante Therapie bzw. auf das Verzichten einer adjuvanten Therapieeskalation klar. Daher muss es Ziel einer chirurgischen Therapie sein, eben nicht nur eine R0-Resektion zu erzielen, sondern diese im besten Fall direkt am Hauptpräparat zu erreichen und nicht erst durch Nachresektionen. Dies muss der Kopf-Hals-Chirurg in seine präoperative Planung mit einbeziehen und ggf. einen größeren bzw. übersichtlicheren Zugang in Betracht ziehen oder ggf. von einem operativen Konzept absehen. Johnson et al. postulierten in einer Kohorte von Patienten mit Mundhöhlenkarzinomen, dass der Kopf-Hals-Chirurg 8–10 mm makroskopisch gesundes Gewebe in alle Richtungen mitresezieren muss, um einen histologischen tumorfreien R‑Status von 5 mm zu erhalten [18]. Dies ist insbesondere zu berücksichtigen, da in dieser Kohorte v. a. in der Gruppe der Patienten mit R1-Resektion der tiefe Absetzungsrand problematisch war. Ong et al. zeigten in einer Kohorte von Patienten mit Zungenkarzinomen, dass Patienten, die durch eine temporäre transmandibuläre Resektion behandelt wurden, seltener eine R1-Resektion am Hauptpräparat aufwiesen als Patienten, bei denen eine transorale Resektion erfolgte. In der Gruppe der Patienten mit transoral resezierten Tumoren stieg somit die Rate für Lokalrezidive, und es sank konsekutiv das RFÜ und die 5‑Jahres-ÜR im Vergleich zur Patientengruppe mit transmandibulär resezierten Tumoren [19]. In der vorliegenden Studie zeigte sich kein signifikanter Unterschied in der Wahl der chirurgischen Zugänge zwischen den verschiedenen Resektionsstatus. Allerdings waren die Subgruppen z. T. auch sehr klein, sodass es nicht möglich ist, abschließend ein Urteil abzugeben.

In diese Studie wurden Patienten mit OPSCC aus den Jahren 2001 bis 2011 eingeschlossen. Zu dieser Zeit wurden weder routinemäßige p16- noch HPV-Nachweise an den Biopsaten oder Operationspräparaten durchgeführt. Daher war eine Stratifizierung nach HPV-Status in dieser Kohorte nicht möglich. Sicher ist dies ein interessanter und relevanter Aspekt, der in zukünftigen Auswertungen Berücksichtigung finden muss. Dennoch haben die Ergebnisse dieser Studie auch in gewisser Weise Bedeutung für die HPV-assoziierten OPSCC. Die Patienten mit HPV-assoziierten OPSCC sind deutlich jünger und gesünder als Patienten mit OPSCC ohne HPV-Assoziation. Zwar sind die HPV-assoziierten OPSCC aggressiver, haben aber eine bessere Prognose [20]. Aufgrund des Alters und der Prognose erleben diese Patienten die volle Wirkung der Spättoxizität einer adjuvanten Therapie, sodass bei der Therapieentscheidung auch diese Faktoren mit Einfluss nehmen und Berücksichtigung finden sollten. Bisher werden HPV-assoziierte OPSCC aufgrund fehlender Evidenz wie die HPV-negativen OPSCC behandelt. Jegliche Modifizierung der Therapie bei HPV-assoziierten OPSCC sollte nur im Rahmen von Studien erfolgen. Einige laufen bereits. Deren Ergebnisse bleiben noch abzuwarten. Natürlich weisen nicht nur die konservativen Therapiekonzepte Spättoxizitäten auf, invasivere chirurgische Maßnahmen haben ebenfalls eine Morbidität für die Patienten, die nicht zu vernachlässigen ist. Hararah et al. erstellten in einer Studie Nomogramme für HPV-positive und HPV-negative OPSCC, um ECE und positive Resektionsränder vorherzusagen zu können und somit den Kopf-Hals-Chirurgen und den Patienten eine Hilfestellung in der Therapieentscheidungsfindung in die Hand zu geben [21]. Allerdings müssen auch diese Daten in größeren Kohorten prospektiv verifiziert werden.

Eine spezielle Situation in dieser Kohorte stellte die Rx-Situation dar. Es zeigte sich bei einer Rx-Situation ein RFÜ von 59 Monaten, was einem RFÜ wie einer R1-Resektion entsprach. Bei der ÜR ergab sich bei Rx-Resektion sogar mit 45 Monaten eine tendenziell schlechtere ÜR als bei R1-Resektion. Die Einflussfaktoren, die zu einer Rx-Situation führen, sind vielfältig. Zum einen ist die anatomische Situation hochkomplex. Zum anderen spielen die Erfahrung des Kopf-Hals-Chirurgen und des Pathologen sowie die Kommunikation zwischen diesen beiden eine relevante Rolle. Auch diese Daten bekräftigen eine R0-Resektion am Hauptpräparat, da diese Situation für alle Beteiligten am eindeutigsten ist, um über eine adjuvante Therapie zu entscheiden bzw. auf eine adjuvante Therapieeskalation zu verzichten.

Überraschend bei der Evaluation der Therapie in dieser Kohorte war, dass der Anteil der Patienten, die eine adjuvante Radiochemotherapie erhielten, von 31 % bei Patienten mit R0-Resektion auf 47 % bei Patienten mit R0-Resektion durch Nachresektion anstieg und weiter auf 64 % bei Patienten mit R1-Resektion und 67 % bei Patienten mit Rx-Resektion. Bereinigt von den Patienten, die eine Therapieeskalation durch ECE erhalten hatten, zeigte sich immer noch eine Therapieeskalation mittels Radio- oder Radiochemotherapie von 17 % bei Patienten mit R0-Resektion am Hauptpräparat, von 35 % bei Patienten mit R0-Resektion durch Nachresektion, von 45 % bei Patienten mit R1-Resektion und von 33 % bei Patienten mit Rx-Resektion. Gerade bei Patienten mit Rx-Situation war die Therapieeskalation zwar häufig durch ECE indiziert, dennoch erhielt ein veritabler Anteil eine Therapieeskalation aufgrund des unklaren R‑Status. Bei Patienten mit R0-Resektion am Hauptpräparat waren die Therapieeskalationen nötig, wenn die Resektionsränder zu knapp waren. Bei den Patienten mit R0-Resektion durch Nachresektion waren zum einen die Resektionsränder trotz Nachresektion entweder zu knapp oder Chirurg und Pathologe waren sich letztendlich nicht sicher, ob tatsächlich ein R0-Status vorlag, sodass die Therapie eskaliert wurde. Daher gilt es, die Therapieeskalation aufgrund einer R0-Resektion durch Nachresektion und einer Rx-Resektion als Kopf-Hals-Chirurgen bei chirurgischen Eingriffen weiter zu minimieren.

## Fazit für die Praxis

Der Kopf-Hals-Chirurg muss bei Patienten mit OPSCC, die für eine operative Sanierung infrage kommen, im Hinblick auf ÜR und RFO eine R0-Resektion am Hauptpräparat anstreben, um adjuvante Therapieeskalationen aufgrund eines unklaren R‑Status zu vermeiden.

## References

[1] Bray F, Ferlay J, Soerjomataram I, Siegel RL, Torre LA, Jemal A (2018). Global cancer statistics 2018: GLOBOCAN estimates of incidence and mortality worldwide for 36 cancers in 185 countries. CA Cancer J Clin.

[2] Berrino F, Esteve J, Coleman MP, Berrino F (1995). Basic issues in estimating and comparing the survival of cancer patients. Survival of cancer patients in europe: the EUROCARE study.

[3] Chi AC, Day TA, Neville BW (2015). Oral cavity and oropharyngeal squamous cell carcinoma—an update. CA Cancer J Clin.

[4] Mehanna H, Beech T, Nicholson T, El-Hariry I, McConkey C, Paleri V (2013). Prevalence of human papillomavirus in oropharyngeal and nonoropharyngeal head and neck cancer—systematic review and meta-analysis of trends by time and region. Head Neck.

[5] Chaturvedi AK, Anderson WF, Lortet-Tieulent J, Curado MP, Ferlay J, Franceschi S (2013). Worldwide trends in incidence rates for oral cavity and oropharyngeal cancers. J Clin Oncol.

[6] Bernier J, Cooper JS, Pajak TF, van Glabbeke M, Bourhis J, Forastiere A (2005). Defining risk levels in locally advanced head and neck cancers: a comparative analysis of concurrent postoperative radiation plus chemotherapy trials of the EORTC (#22931) and RTOG (# 9501). Head Neck.

[7] Kurita H, Nakanishi Y, Nishizawa R, Xiao T, Kamata T, Koike T (2010). Impact of different surgical margin conditions on local recurrence of oral squamous cell carcinoma. Oral Oncol.

[8] Woolgar JA, Scott J, Vaughan ED, Brown JS, West CR, Rogers S (1995). Survival, metastasis and recurrence of oral cancer in relation to pathological features. Ann R Coll Surg Engl.

[9] Sessions DG, Spector GJ, Lenox J, Parriott S, Haughey B, Chao C (2000). Analysis of treatment results for floor-of-mouth cancer. Laryngoscope.

[10] Eldeeb H, Macmillan C, Elwell C, Hammod A (2012). The effect of the surgical margins on the outcome of patients with head and neck squamous cell carcinoma: single institution experience. Cancer Biol Med.

[11] Cooper JS, Pajak TF, Forastiere AA, Jacobs J, Campbell BH, Saxman SB (2004). Postoperative concurrent radiotherapy and chemotherapy for high-risk squamous-cell carcinoma of the head and neck. N Engl J Med.

[12] Bernier J, Domenge C, Ozsahin M, Matuszewska K, Lefebvre JL, Greiner RH (2004). Postoperative irradiation with or without concomitant chemotherapy for locally advanced head and neck cancer. N Engl J Med.

[13] Hunter KU, Schipper M, Feng FY, Lyden T, Haxer M, Murdoch-Kinch CA (2013). Toxicities affecting quality of life after chemo-IMRT of oropharyngeal cancer: prospective study of patient-reported, observer-rated, and objective outcomes. Int J Radiat Oncol Biol Phys.

[14] Guo GZ, Sutherland KR, Myers C, Lambert P, Loewen SK, Quon HC (2016). Prospective swallowing outcomes after IMRT for oropharyngeal cancer: dosimetric correlations in a population-based cohort. Oral Oncol.

[15] Eisbruch A, Kim HM, Feng FY, Lyden TH, Haxer MJ, Feng M (2011). Chemo-IMRT of oropharyngeal cancer aiming to reduce dysphagia: swallowing organs late complication probabilities and dosimetric correlates. Int J Radiat Oncol Biol Phys.

[16] Wong ATT, Lai SY, Gunn GB, Beadle BM, Fuller CD, Barrow MP (2017). Symptom burden and dysphagia associated with osteoradionecrosis in long-term oropharynx cancer survivors: a cohort analysis. Oral Oncol.

[17] Eraj SA, Jomaa MK, Rock CD, Mohamed ASR, MD Anderson Head and Neck Cancer Symptom Working Group (2017). Long-term patient reported outcomes following radiation therapy for oropharyngeal cancer: cross-sectional assessment of a prospective symptom survey in patients ≥65 years old. Radiat Oncol.

[18] Johnson RE, Sigman JD, Funk GF, Robinson RA, Hoffman HT (1997). Quantification of surgical margin shrinkage in the oral cavity. Head Neck.

[19] Ong HS, Gokavarapu S, Tian Z, Li J, Cao W, Zhang CP (2018). Does a mandibular access osteotomy improve survival in pT2 oral tongue cancers? Retrospective study at a single institution. Int J Oral Maxillofac Surg.

[20] Ang KK, Harris J, Wheeler R, Weber R, Rosenthal DI, Nguyen-Tan PF (2010). Human papillomavirus and survival of patients with oropharyngeal cancer. N Engl J Med.

[21] Hararah MK, Stokes WA, Jones BL, Oweida A, Ding D, McDermott J (2018). Nomogram for preoperative prediction of nodal extracapsular extension or positive surgical margins in oropharyngeal squamous cell carcinoma. Oral Oncol.

